# Screening for osteo-renal involvement in the Romanian HIV cohort

**DOI:** 10.1186/1471-2334-14-S7-P65

**Published:** 2014-10-15

**Authors:** Anca Streinu-Cercel, Oana Săndulescu, Claudiu Mihai Șchiopu, Cristiana Oprea, Sorin Rugină, Carmen Mihaela Dorobăț, Voichița Lăzureanu, Felicia Constandiş, Corina Itu, Augustin Cupşa, Carmen Chiriac, Adrian Streinu-Cercel

**Affiliations:** 1 Carol Davila University of Medicine and Pharmacy, Bucharest, Romania; 2 National Institute for Infectious Diseases "Prof. Dr. Matei Balş", Bucharest, Romania; 3 Clinical Hospital of Infectious and Tropical Diseases “Dr. Victor Babeş”, Bucharest, Romania; 4 Clinical Infectious Diseases Hospital of Constanța, Romania; 5 Faculty of Medicine, “Ovidius” University, Constanța, Romania; 6 Infectious Diseases Hospital “Sf Parascheva”, Iaşi, Romania; 7 “Gr.T.Popa” University of Medicine and Pharmacy, Iaşi, Romania; 8 Dr. Victor Babeş University of Medicine and Pharmacy, Timişoara, Romania; 9 Infectious Diseases Hospital, Braşov, Romania; 10 Clinical Hospital of Infectious Diseases, Cluj-Napoca, Romania; 11 “Victor Babeş” Clinical Hospital of Infectious Diseases and Pneumology, Craiova, Romania; 12 Clinical County Hospital Mureş, Romania

## Background

When assessing comorbidities in HIV-infected patients, the bone and the kidney represent important target organs that can potentially be affected by both virus and antivirals. Given the particular characteristics of the Romanian HIV cohort [1], most of the patients have experienced HIV infection in childhood and have received multiple therapeutic regimens since the advent of antiretroviral (ARV) therapy. Thus, the need to screen for osteo-renal impairment in these patients is high on the priority list [2].

## Methods

We have started a project to evaluate key markers of kidney disease and assess the risk for fracture and kidney involvement in the Romanian cohort of HIV-infected patients.

## Results

To date, 645 subjects have been enrolled, the group being representative for the whole country, being monitored in all the 9 regional HIV/AIDS reference centers in Romania. We present the descriptive data for this group of patients.

The mean age was 24.3±2.4 years, with a median age at HIV diagnosis of 11 years old. The main transmission route for HIV infection was parenteral (75.35%). Vertical transmission accounted for 1.4% of cases, heterosexual contact for 6.05%, homosexual contact for 0.61% and in 16.59% of cases the transmission route could not be ascertained.

The current median CD4 cell count was 488 cells/cmm, with a median nadir CD4 cell count of 110 cells/cmm. Most of the patients had received multiple ARV regimens over time: 50.85% over 3 regimens, 47.13% 1-3 regimens, and only 2.02% were ARV-naïve.

## Conclusion

In the following months we plan to complete the osteo-renal evaluation for the patients with the characteristics described above, and to develop a clinical algorithm for predicting, diagnosing and monitoring bone and kidney involvement in patients with chronic viral infections.
